# Generating 3D Multispectral Point Clouds of Plants with Fusion of Snapshot Spectral and RGB-D Images

**DOI:** 10.34133/plantphenomics.0040

**Published:** 2023-04-03

**Authors:** Pengyao Xie, Ruiming Du, Zhihong Ma, Haiyan Cen

**Affiliations:** ^1^ College of Biosystems Engineering and Food Science, Zhejiang University, Hangzhou 310058, China.; ^2^ Key Laboratory of Spectroscopy Sensing, Ministry of Agriculture and Rural Affairs, Hangzhou 310058, China.

## Abstract

Accurate and high-throughput plant phenotyping is important for accelerating crop breeding. Spectral imaging that can acquire both spectral and spatial information of plants related to structural, biochemical, and physiological traits becomes one of the popular phenotyping techniques. However, close-range spectral imaging of plants could be highly affected by the complex plant structure and illumination conditions, which becomes one of the main challenges for close-range plant phenotyping. In this study, we proposed a new method for generating high-quality plant 3-dimensional multispectral point clouds. Speeded-Up Robust Features and Demons was used for fusing depth and snapshot spectral images acquired at close range. A reflectance correction method for plant spectral images based on hemisphere references combined with artificial neural network was developed for eliminating the illumination effects. The proposed Speeded-Up Robust Features and Demons achieved an average structural similarity index measure of 0.931, outperforming the classic approaches with an average structural similarity index measure of 0.889 in RGB and snapshot spectral image registration. The distribution of digital number values of the references at different positions and orientations was simulated using artificial neural network with the determination coefficient (*R*^2^) of 0.962 and root mean squared error of 0.036. Compared with the ground truth measured by ASD spectrometer, the average root mean squared error of the reflectance spectra before and after reflectance correction at different leaf positions decreased by 78.0%. For the same leaf position, the average Euclidean distances between the multiview reflectance spectra decreased by 60.7%. Our results indicate that the proposed method achieves a good performance in generating plant 3-dimensional multispectral point clouds, which is promising for close-range plant phenotyping.

## Introduction

High-throughput plant phenotyping that refers to the quantitative assessment of various plant traits has been considered as an important tool for supporting plant breeding and precision farming. While robust and high-precise plant phenotyping is still a great challenge due to the complexity of the plant structures and phenotypic dynamics over the growth period. Recently, different optical sensing-based high-throughput phenotyping systems for the laboratory, greenhouse, and infield have been developed. Among them, multi/hyperspectral imaging is considered as a promising technique to acquire both spectral and spatial information simultaneously [1,2]. Various phenotypic traits such as physiological, biochemical, morphological, and performance ones could be modeled with extracted image features [3]. However, because of the limitation of imaging dimensions, factors such as inclination and curling of plant leaves, mutual occlusion between plant leaves and other organs or canopy, and plant–illumination interaction could greatly affect the integrity and accuracy of spectral imaging for plants. Three-dimensional (3D) data, describing the spatial information of the target, is widely used for plant phenotyping [4]. 3D data can be acquired through time-of-flight techniques, such as lidar, laser scanners, and depth cameras, or stereo vision techniques, such as binocular cameras and multiview cameras [5,6]. Since spectral images and 3D data are highly complementary in presenting information about plant growth and development, analyzing on the fusion of multimodal data (data acquired by different sensors) can provide insights into high-throughput plant phenotyping. 3D multispectral point cloud, representing both the coordinates of discrete points and multiband spectral response, is a promising form of multimodal data [7]. However, generating high-quality 3D multispectral point clouds at close range that usually provides a submillimeter or millimeter spatial resolution remains challenging [8]. Behmann et al. [9] applied 3D hyperspectral models to sugar beet plants, and the detection accuracy was improved with reducing the overestimated infection rate of 15% compared to only using hyperspectral images. Sun et al. [10] used multispectral 3D imaging to predict relative chlorophyll content of tomato plants, and the performance of the prediction model developed from 3D multispectral point cloud was better than those of the single-view point cloud model. Although these approaches have shown the potential for accurate plant phenotyping, the accuracy of generated 3D multispectral point clouds is still relatively low because of the complexity of multimodal data fusion and the illumination effect [11]. Multimodal data fusion refers to achieving complementarity of different feature sets of data acquired from the same object under different perspectives or domains. Illumination effects refer to illumination inhomogeneity in the image caused by the interaction of uneven light distribution and complex plant structures. These 2 factors will be elaborately discussed in the following 2 paragraphs.

Generating 3D multispectral point clouds of plants can be divided into direct and indirect methods. The direct method obtains the spectral and spatial information through the intensity of reflected light and time-of-flight techniques, respectively. Since the signals come from the same sensor, this method does not require data registration or fusion. Hu et al. [12] proposed a hyperspectral light detection and ranging (LiDAR) technique by combining hyperspectral and LiDAR sensors to directly generate 3D multispectral point clouds. However, this application of LiDAR combined with small target objects has little applicability to the whole-plant scale. The indirect method obtains 3D multispectral point clouds by fusing images of multispectral camera with the 3D point cloud reconstructed from 3D sensors [13,14]. Specifically, plant spectral and spatial information are registered into the same image coordinate system and assigned to the same pixels. Several studies have demonstrated the potential of multimodal image registration algorithms such as structure-from-motion and multiview stereo and enhanced correlation coefficient (ECC) for fusion of unmanned aerial vehicle images [15,16]. Furthermore, scale-invariant feature transform algorithm was used to mosaic low-resolution unmanned aerial vehicle images for crop growth monitoring and achieved the structural similarity index of 0.9 [17]. When handling the close-range image registration, the heterogeneity of corresponding pixels and the geometric distortion in 2 images acquired at close range could be amplified with the smaller field of view, thus reducing the accuracy of image registration. Therefore, it is necessary to develop a new registration algorithm that can achieve both accuracy and efficiency for multimode image registration suitable for close-range imaging.

In addition, close-range imaging registration could be influenced by illumination effects. Nonuniformity of light source is one of the factors that lead to the illumination inhomogeneity in the image field of view [18]. The self-occlusion of the plant organs and multiple scattering among dense canopies can cause nonlinearity in Lambert–Beer’s relation between the optical density and the chemical concentration, thereby reducing the accuracy of predicting physiological and biochemical traits [19]. Liang et al. [20] made the first attempt to reconstruct 3D hyperspectral plant models from hyperspectral images. However, the single frames of plant 3D multispectral point clouds obtained from different views could not be directly registered into a complete model because of the difference in the illumination condition under different viewing angles and the complexity of plant geometries. Elimination of illumination effect is necessary to obtain accurate spectral information for close-range spectral images in plant phenotyping. The conventional method to obtain spectral reflectance is to calculate the percentage of digital number (DN) values acquired from raw images of the plant and the reference with the baseline correction. The reference (usually flat) needs to be kept at the same position and orientation, with the poses of cameras remaining stable to ensure the same illumination condition during image acquisition. This method fails in close-range imaging because it is impossible to match all poses of leaves or other organs by changing the position or orientation of the flat reference under nonuniform light distribution on plants. Standard normal variate (SNV) and its variants are widely used in spectral analysis to eliminate the multiplier term of the illumination effects in Lambert's reflection [21]. However, SNV has serious drawbacks in terms of physical interpretation and robustness of estimation models, as it assumes that all spectral wavelengths are affected similarly. Thus, all the variables in SNV model are equally considered, resulting in a nonuniform correction across all bands. Mechanistic methods such as various radiative transfer models have also been proposed to solve the interaction between incident light and canopies. PROSPECT model is one of the most popular radiative transfer models based on leaf directional–hemispherical reflectance factor and transmittance factor spectra. Its variant, PROCOSINE, a model combining PROSPECT and ClOse-range Spectral ImagiNg of lEaves (COSINE) is viewed as a promising physics-based model to describe leaf specular reflection and local leaf orientation [8,22,23]. However, the applications of the PROCOSINE model are limited to leaf scale. As for implementing PROCOSINE for a whole plant at close range, the performance of PROCOSINE model decreased because of the difficulty in obtaining accurate illumination parameters, plant morphological, and structural parameters required by the model. The confounding effects between the incident angle and structure parameter could also be a reason causing unsatisfactory results of PROCOSINE model in the visible and near-infrared region. Considering the problems above, we therefore adopted the stereo reference for reflectance correction. Compared with the flat reference, the stereo reference has more structural features, which can be easily used to replace flat reference placed at different positions and orientations. Zhang et al. [24] first demonstrated the effectiveness of using a stereo reference to correct the corresponding original image. However, the design of their stereo reference was complex, and its application was limited to the leaf scale, where the illumination conditions among different areas of the same leaf were considered approximately identical. In addition, the limited 3D light field characterization of their stereo reference and the sparse sampling of the reflectance of reference also adversely affected its application to complex individual plants and canopies.

Therefore, this study aims to develop a new method for generating high-quality 3D multispectral point clouds of plants. The specific objectives of this study are (a) to develop a new multimodal image registration method coupled with both rigid and nonrigid transformation to fuse depth and snapshot spectral images acquired at close range, (b) to design a reflectance correction method based on hemisphere references with artificial neural network (ANN) model, and (c) to compare the proposed method with widely used multimodal image registration methods and search-based reflectance correction method in accuracy and efficiency.

## Materials and Methods

### Experimental setup

**Fig. 1.**
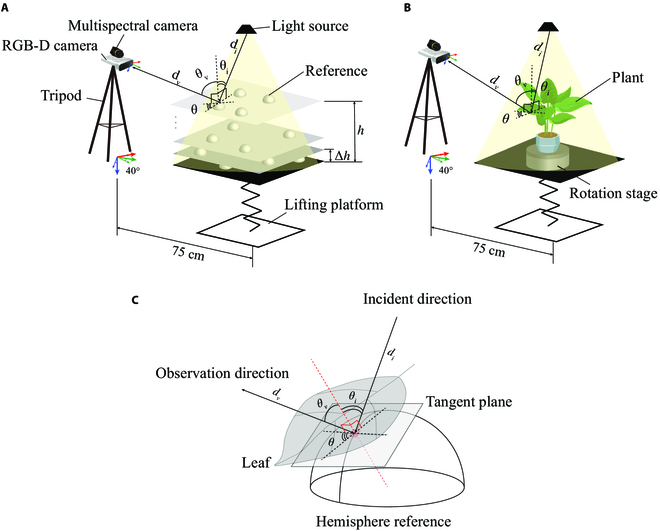
Experimental setup of the imaging system. (A) Schematic diagram of the raw image acquisition system for the hemisphere references. (B) Schematic diagram of the raw image acquisition system for the plant. (C) Simulation of the plant leaf and the hemisphere reference tangent to each other and the relevant 3D light field features. In this case, the plant and the reference at the tangent point share the same 3D light field features. Here, *d_i_* denotes the distance from the point to the light source, *d_v_* denotes the distance from the point to the camera optical center, *θ_i_* denotes the angle between the direction vector of the incident light and the normal vector of the surface where the point is located, *θ_v_* denotes the angle between the observation direction vector and the normal vector of the surface where the point is located, and *θ* denotes the angle between the projection vector of the incident light direction vector and the projection vector of the observation direction vector on the surface where the point is located.

The configuration of the imaging system used to obtain RGB-D and multispectral images is shown in Figure 1. The RGB-D sensor (Azure Kinect DK, Microsoft, Redmond, WA, USA) and the multispectral camera (MQ022MG-CM, XIMEA, Munster, Germany) were fixed together using a self-designed adapter and then mounted on a tripod. A tungsten halogen light source with the power of 60 W was used to generate illumination for the experiment. The individual evergreen plant grown in a pot was placed on the sample handling stage for imaging. RGB-D images with the spatial resolution of 720 × 1280 pixels and multispectral images with 25 bands at 650 to 950 nm and the spatial resolution of 216 × 409 pixels were then acquired from the individual plant. During the image acquisition, the pitch angle of the 2 cameras was fixed at 40°, and the horizontal distance between the tripod and the lifting platform was about 75 cm (Fig. 1B). Four “computer numerical control” machined and surface matte-treated solid Teflon hemispheres with the diameter of 100 mm were made as the references. One image pair obtained in each position of the plant or hemisphere references is consisted of RGB-D image and 25-band multispectral image. To create the reference and sample image databases that could share the enough tangent points of the reference with those of the leaf, reference images were acquired at 70 different positions by changing the vertical position 10 times at the distance of 10% of the plant height with the horizontal position changing 7 times at each vertical position (Fig. 1A). Figure 1C shows the ideal positional relationship between the leaf and the hemisphere reference. Theoretically, the sampling resolution and modeling accuracy could be further improved by changing more positions. However, the pre-experiment results showed that after more than 70 position changes and image acquisitions, the further improvement of the experimental results was not obvious, and it would increase the consumption of labor and computing resources. RGB-D images and multispectral images of the plant were obtained at 15 different views by rotating the sample holder. With such reference and sample image databases, we hypothesized that the reflectance could be directly calculated on the basis of the spectral DN values of plant leaf and hemisphere reference.

### Multimodal image registration

As RGB-D images and multispectral images of plants were acquired from 2 different sensors, it was necessary to perform image registration before data fusion. We accomplished the entire coarse-to-fine registration process by 3 steps. In the first step, segmentation and brightness balancing of these image pairs were implemented to remove the background noise and enhance the image quality. Speeded-Up Robust Features (SURF) was then used as the coarse registration method, followed by the adaptive parameter threshold selection method to achieve the automatic process for the selection of SURF features with the best registration performance [25]. Finally, the nonparametric deformable registration method Demons was used as the fine registration method, which feeds back the registered image rectified by the deformation displacement as a new input for next round iteration until the stopping rule is satisfied [26]. The details of the 3-step registration are described in the following sections.

#### Step 1: Preprocessing for registration

The goal of the first step was to extract the plant region of the image pair prepared for registration. The segmentation methods used for different types of images were not identical because of the different image characteristics. We registered the RGB image and the depth image to make them equivalent in registration with the multispectral image. Hue–saturation–value color space thresholding and depth thresholding were applied to segment the plant region in the RGB-D image. Normalized difference vegetation index thresholding was used to segment the plant region in the multispectral image. Morphological filtering was used to remove the noise from segmented images, so as to obtain pure plant foreground images.

After plant segmentation, it was necessary to balance the brightness of the image pair to reduce the difference of subsequent SURF detection on different images in the same image pair. We chose the G-component grayscale image of the RGB-D image (*I_g_*) and the seventh band (740.7 nm) of the multispectral image ($I_{\mathrm{ms}}^{7}$) as the fixed image and moving image, respectively. Histogram matching was used to change $I_{\mathrm{ms}}^{7}$ to have the same grayscale histogram as *I_g_*, so as to correct the brightness difference.

#### Step 2: Coarse registration by adaptive SURF

The main idea of the second step was to enable moving images to be roughly aligned with fixed images by SURF, reducing fine-registration iterations and improving accuracy. The image pyramids were first built by forming the scale space using the scaled box filters and convolutions. The approximated Hessian determinant *c*(*x*, *y*, *σ*) of *I_g_* and $I_{\mathrm{ms}}^{7}$ were then calculated pixel by pixel:

$$c\left(x,y,\sigma \right)=D_{\mathrm{xx}}D_{\mathrm{yy}}-{\left(wD_{\mathrm{xy}}\right)}^{2}$$ (1)

where *x*, *y*, and *σ* denote the pixel coordinates and the scale, respectively. *D_xx_*, *D_xy_*, and *D_yy_* are the approximate convolution values obtained using the box filter. The relative weight *w* of the filter responses is used to balance the expression for the Hessian’s determinant, which is required for the energy conservation between the Gaussian kernels and the approximated Gaussian kernels. Although the weighting changes depending on the scale, we used the constant value *w* = 0.9 to ensure the approximation of Hessian response based on the reported study [25], which has good computational efficiency and does not significantly affect the accuracy of the determinant approximation.

As the next step, the establishment of SURF database was performed on the basis of the Hessian responses above. The key points {*p*_0_}*_g_* and ${\left\{p_{0}\right\}}_{\mathrm{ms}}^{7}$, which were elements of the database, were initially located by comparing Hessian responses of each pixel and by performing the nonmaximum suppression with 3 × 3 × 3 nearby pixels in the scale space.

Usually, the selection of the SURF threshold was implemented manually to obtain a suitable number of SURF feature point pairs from the database. The best threshold of one image pair differed from others, causing inefficiency during this manual process. Here, we proposed an adaptive thresholding method to search for the best threshold of each image pair automatically. The extreme value of *c*(*x*, *y*, *σ*) in the SURF database was calculated at first. Then, the interval [*c_min_*, *c_max_*] was divided into (*i* + 1) equal parts, and the nodes were taken as (*c*_1_, *c*_2_, …, *c_i_* ). We adopted the Euclidean distance *d* of the eigenvectors of each point pair in {*p*_0_}*_g_* and ${\left\{p_{0}\right\}}_{\mathrm{ms}}^{7}$ as the matching degree. After the distance calculation, the interval [*d_min_*, *d_max_*] was divided into (*j* + 1) equal parts, and the nodes were taken as (*d*_1_, *d*_2_, …, *d_j_* ). Finally, {*p*_0_}*_g_* and ${\left\{p_{0}\right\}}_{\mathrm{ms}}^{7}$ were filtered using all combinations of (*c_i_*, *d_j_*) as thresholds to get {*p*_1_}*_g_* and ${\left\{p_{1}\right\}}_{\mathrm{ms}}^{7}$. Rigid transformation was performed in each iteration. In this step, the transformation matrix was estimated for the point set {*p*_1_}*_g_* and ${\left\{p_{1}\right\}}_{\mathrm{ms}}^{7}$ under (*c_i_*, *d_j_*) threshold. The transformed image ${\left(I_{\mathrm{ms}}^{7}\right)}_{t}$ was obtained using bicubic interpolation. Three image transformation matrices were considered, namely, similarity transformation, projective transformation, and affine transformation. The general formula is as follows:

$$\left[\begin{array}{c} x_{t}\\ y_{t}\\ 1 \end{array}\right]=\left[\begin{array}{ccc} r_{1} & r_{2} & r_{3}\\ r_{4} & r_{5} & r_{6}\\ r_{7} & r_{8} & r_{9} \end{array}\right]\left[\begin{array}{c} x\\ y\\ 1 \end{array}\right]=T\left[\begin{array}{c} x\\ y\\ 1 \end{array}\right]$$ (2)

Updating and stopping rules for adaptive thresholding were given as follows.

1) Updating rule*:* The proposed updating rule was based on the order of structural similarity index measure (SSIM) between ${\left(I_{\mathrm{ms}}^{7}\right)}_{t}$ and *I_g_* under all thresholds. The threshold indices of the first 4 SSIMs were searched and were then combined into new intervals [*c_m_*, *c_n_*] and [*d_p_*, *d_q_*].
2) Stopping rule: The stopping rule was determined by the reduction threshold of SSIM. The iterations would be repeated until $\frac{{\text{SSIM}}_{\text{step}}-{\text{SSIM}}_{\text{step}-1}}{{\text{SSIM}}_{\text{step}-1}}\leq e_{1}$. The ${\left(I_{\mathrm{ms}}^{7}\right)}_{t}$ and *T_final_* corresponding to the best SSIM was reserved.

#### Step 3: Fine registration by Demons

The third step performed nonrigid transformation for the best-registered image from the second step. After repeating the preprocessing step to balance the brightness of ${\left(I_{\mathrm{ms}}^{7}\right)}_{t}$, we used Thirion’s rule of Demons [27] to calculate the deformation displacement:

$$u=\frac{s-m\circ t}{{\left\|\nabla m\right\|}^{2}+\alpha^{2}{\left(s-m\circ t\right)}^{2}}\nabla m$$ (3)

where *s* is the fixed image *I_g_*, *m* is the moving image ${\left(I_{\mathrm{ms}}^{7}\right)}_{t}$, *t* is the deformation displacement, and *u* is the increment of *t* during each iteration. ∘ represents the transformation of the image.

A stopping rule was also designed to determine when the iterative process should be terminated. In this step, the calculation of the SSIM was between ${\left(I_{\mathrm{ms}}^{7}\right)}_{t}$ and *I_g_*. The iterations would be repeated until $\frac{{\text{SSIM}}_{\text{step}}-{\text{SSIM}}_{\text{step}-1}}{{\text{SSIM}}_{\text{step}-1}}\leq e_{2}$. The deformation displacement *t_final_* corresponding to the best SSIM was reserved.

Finally, the coarse-to-fine deformation matrix was the combination of *T_final_* and *t_final_*. The fine-registered 25-band multispectral image could be obtained by applying both matrix to $\left\{I_{\mathrm{ms}}^{b}\;|\;b=1,2,\ldots ,25\right\}$.

### 3D light field feature extraction

Bidirectional reflectance distribution function (BRDF) is a commonly used physical model describing the relationship between the incident and the reflected light on a surface. The case where the surface is a general or isotropic material can be respectively described as *f*(*θ_i_*, *ϕ_i_*, *θ_o_*, *ϕ_o_*) or *f*(*θ_i_*, *θ_o_*, *ϕ*). Here, *θ_i_* and *θ_o_* are zenith angles of the incident light and the observation directions, *ϕ_i_* and *ϕ_o_* are azimuthal angles of the incident light and the observation directions, and *ϕ* denotes the angle between the projection of the incident light direction vector and the projection of the observation direction vector on the surface. Inspired by the parametrization of the BRDF model where plant leaves are considered isotropic ideal Lambertian models, we selected the distance, the angle between the incident and observation directions, and the 3D coordinates as the reflection-related 3D light field features. These features were used to determine the position and the orientation toward the light source and cameras for a small region of the plant or hemisphere reference. In addition to the spatial position represented by *xyz*, 5 other 3D light field features were also used for relative reflectance calculation. The details of these 5 features are presented in Fig. 1C.

**Fig. 2.**
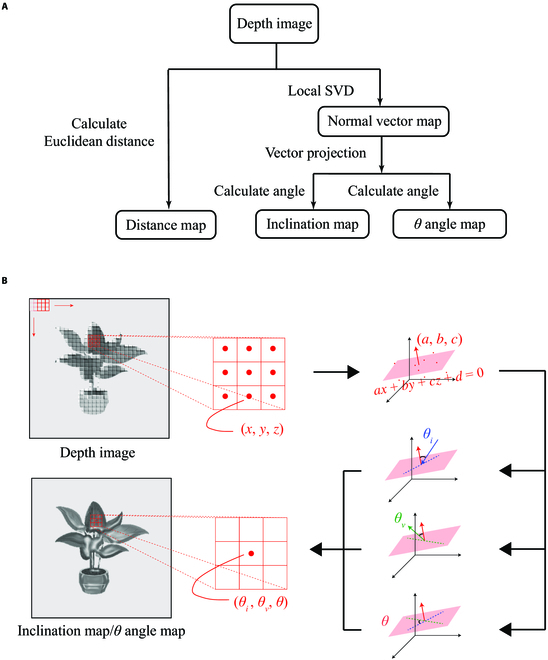
Extraction of plant 3D light field features. (A) Procedure of feature extraction. In this procedure, distance map was calculated using Euclidean distance from the depth image. The intermediate step in the calculation of angle map such as inclination map and *θ* map is the generation of the normal vector map using local SVD. Then, the corresponding angle values were calculated by means of vector projection. (B) Calculation of the normal vector and included angle of each point in the depth image pixel by pixel. The local SVD was performed to fit the plane of all points contained in each 3 × 3 sliding window. Each type of angle can be calculated on the basis of the normal vector of the plane. The results were stored as multichannel images without altering the pixel position.

Compared to unorganized frames of point clouds, the use of the whole depth images helped to establish pixel correspondences with registered multispectral images and facilitates the use of sliding window. To calculate the 3D light field features, local point cloud was converted from the corresponding region of depth image by reshaping. We used local singular value decomposition (SVD) to fit the plane of all points contained in each 3 × 3 sliding window and calculated the normal vector of the point cloud as the normal vector of the plane where the center point of the sliding window was located. At last, the angles between the normal vector and the other vectors in Fig. 2 were calculated.

### ANN modeling

**Fig. 3.**
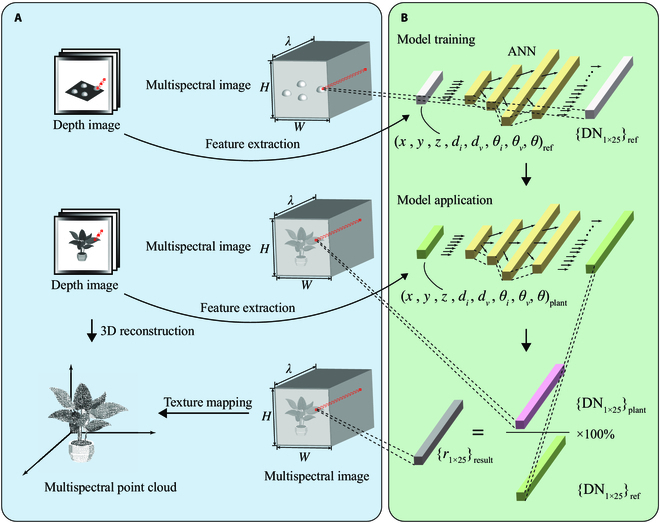
Principle and flow chart of plant multispectral reflectance correction. (A) The flow chart of generating plant multispectral point cloud. Raw images such as depth image and multispectral image were registered, and multispectral image was reshaped as a multichannel image at the beginning of the procedure. Then, follow the point cloud generation that relies on the transformation from depth image coordinate system to the world coordinate system under the constrains of the camera intrinsic parameters. Finally, with the fusion of multiview point clouds and the mapping of corrected multispectral textures, the 3D multispectral point cloud model was constructed. (B) The flow chart of calculating the spatial distribution of the DN values of the references and correcting the plant spectral reflectance using ANN. In the stage of model training, the 3D light field features of references were extracted from depth image as independent variables and the spectral DN values as dependent variables. In the stage of model application, the 3D light field features of plant were set as input to obtain the predictions of the corresponding DN values of the reference. Finally, the reflectance image is corrected pixel by pixel based on this method to generate a mappable texture.

We designed an ANN model to directly establish relationships between 3D light field features and spectral DN values of the references instead of painstakingly obtaining tabulated BRDF. The 3D light field features were extracted as independent variables, and the spectral DN values were obtained as dependent variables. The input and output layer sizes of ANN were determined by the number of 3D light field features and the number of spectral bands, respectively. The number of neurons in the middle layer was set to 20 according to the empirical formula and the preliminary experiment. In the training stage, the input and the output were the combinations of 3D light field features extracted from the depth image of the hemisphere references and the 25-band spectral DN values. In the application stage, the combinations of 3D light field features extracted from the depth image of the plant were input to predict the corresponding 25-band spectral DN values of the reference. The corrected multispectral reflectance image of the plant was then obtained by calculating the percentage ratio of DN values between the plant and the reference pixel by pixel. Finally, this correction result was mapped to the 3D point cloud of the plant (reshaped from the whole depth image) as a texture to generate a single frame of 3D multispectral point cloud. Figure 3 presents the principle and flow chart of this correction.

We used 3 specific feature combinations as inputs to the model for comparison: 8 inputs (*d_i_*, *d_v_*, *θ_i_*, *θ_v_*, θ, *x*, *y*, *z*), 5 inputs (*d_i_*, *d_v_*, *θ_i_*, *θ_v_*, θ), and 3 inputs (*x*, *y*, *z*). The dataset size was the same as the total number of pixels of the segmented image, which was 911,120. The dataset was divided into training set, validation set, and test set by 70%:15%:15%, and the training error was calculated by mean squared error. We selected a larger number of training epochs to ensure the convergence of the model. Because of the scale heterogeneity of the data, we applied minimum–maximum normalization before model training.

### Model comparison

In addition to the ANN model, we also adopted other widely used regression models such as linear regression, decision trees, random forest, support vector machines, and Gaussian process regression. We compared the fitting performance of these models on the dataset. The models were all trained with their respective optimal parameters and kernel functions, and 5-fold cross-validation was used.

There are 2 methods for obtaining the DN spectra of the references at specific 3D light field features. One is to train a model for direct prediction, and the other is to build a DN spectra database for search. The search-based method creates a bidirectional match between a certain 3D light field feature vector of plants and the closest vector in the database for query. Since there are many multidimensional vector distance metrics, the matching results also vary, making it hard to be generalized. In addition, the matching time depends on the time complexity of the searching algorithm and the data volume of the database. However, using general search algorithms to solve the large data volume in our scenario may result in unacceptable matching times. To further compare the performance of the model-based method and search-based method, we selected several common distance metrics (e.g., Euclidean, Cosine, Mahalanobis, and Chebychev) and modified distance metrics (e.g., weighted Euclidean, Cosine after principal components analysis, and weighted Mahalanobis) for the search. We simulated 2 DN distributions under the independent variable control conditions based on the 5-input ANN model-based method and the search-based method with the best distance metric and binary sorting tree [time complexity *O*(log *n*)].

### Validation experiment

We presented an experiment to verify the performance of our proposed reflectance correction model. To collect data for comparison, a whiteboard with a reflectivity of 0.99 was placed flat at the average height of the plant canopy, and the multispectral images of the plant and the whiteboard were collected successively without changing the camera pose. Following Al Makdessi et al. [28], the measured value of the ASD spectrometer (Analytical Spectral Device Inc., Boulder, CO, USA) equipped with the ASD leaf clip was used as the ground truth for reference. In this study, 12 leaf positions were selected in terms of their chlorophyll content, canopy height, and leaf inclination. We compared the error between the spectral reflectance curves before and after reflectance correction and the true spectral reflectance curves of all the sampled leaf positions in 15 different placements in the dataset. As for evaluation metrics, we selected the root mean squared error (RMSE) of the spectra before and after reflectance correction and the Euclidean distance range (the difference between the maxima and minima) between pairs of multispectral reflectance curves obtained from all viewpoints belonging to a certain leaf position:

$$\text{RMSE}=\frac{1}{N}\sum_{v=1}^{N}\sqrt{\frac{1}{M}\sum_{b=1}^{M}{\left(s_{v}^{b}-s_{\mathrm{gt}}^{b}\right)}^{2}}$$ (4)

$$\text{Range}=\text{max}\left(\sqrt{\frac{1}{M}\sum_{b=1}^{M}{\left(s_{m}^{b}-s_{n}^{b}\right)}^{2}}\right)$$ (5)

where $s_{v}^{b}$ is the reflectance value of viewpoint *v* at band *b*, $s_{\mathrm{gt}}^{b}$ is the reflectance value measured by ASD at band *b*, *M* is the number of bands, and *N* is the number of viewpoints, *m*, *n* ∈ [1, *N*].

## Results

### Performance of image registration

**Fig. 4.**
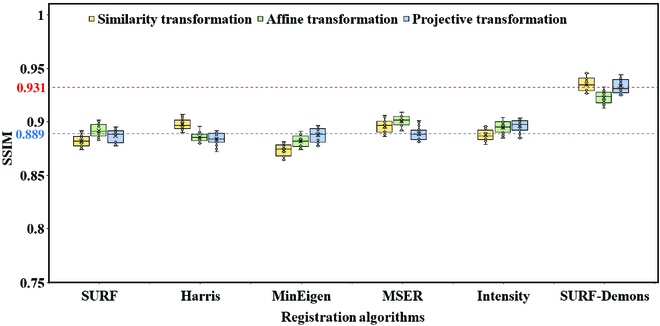
Performance comparison of the different registration algorithms and transformation methods.

Figure 4 presents the comparison of different registration algorithms such as SURF, Harris, MinEigen, maximally stable extremal regions (MSER), and intensity-based registration and transformation methods such as similarity, affine, and projective transformation. The average SSIM value of the SURF-Demons joint registration algorithm was 0.931 with all the datasets, which was higher than those obtained from the classic image registration algorithms (0.889 on average). According to this average value, the registration performance was improved by 4.72%. The difference of SSIM under different transformation methods was not significant, and there was no optimal transformation method suitable for all image registration algorithms. As shown in Table 1, the average processing time of the SURF-Demons joint registration algorithm for a single image was 1.93 s, similar to the performance of the intensity-based registration algorithm (1.37 s) and higher than other feature-based registration algorithms (less than 0.03 s). It reveals that compared to the feature-based registration algorithms, Demons algorithm has higher computational complexity. In summary, SURF-Demons algorithm achieves the best registration accuracy of close-range images with an acceptable processing time.

**Table 1.** Efficiency comparison of various registration algorithms.

Evaluation metric	Models	Transformation methods		
		Similarity	Affine	Projective
Processing time per image(s)	SURF	0.02	0.02	0.02
	Harris	0.02	0.02	0.02
	MinEigen	0.02	0.02	0.03
	MSER	0.01	0.01	0.01
	Intensity	1.37	1.36	1.36
	SURF-Demons	1.93	1.92	1.93

### Comparison of different models

Table 2 shows the regression results of several commonly used models evaluated by metrics of *R*^2^ and RMSE. These regression models were run with statistics and machine learning toolbox (v11.7) in MATLAB 2020a software (MathWorks, Natick, MA, USA) on a computer with Intel Core i7-9850H @ 2.60 GHz, Windows 10 64-bit operating system, and 64 GB of ECC RAM. As the model input number increased, the fitting accuracy of all models improved. When the input features were few, the ANN model exhibited better interpretability and achieved lower error. In the case of many input features, the ANN model achieved the lowest error under the condition that all models generally achieved better interpretability. Compared with the fluctuation of measured reflectivity (usually greater than 0.1) due to the change of height and pose during the conventional whiteboard calibration process, this error was acceptable in practical applications. ANN has been shown to outperform other regression models when the number of samples is large. It is universally acknowledged that the network depth and the number of nodes could be increased to further improve the model prediction result. However, the marginal improvement of the model performance at this level was limited, and it would consume more computing resources. Therefore, there was no need to continue optimization.

**Table 2.** Fitting results of different regression models between features and normalized DN values of the reference.

Model	Evaluation metrics	Number of input features		
		3	5	8
LR	*R* ^2^	0.59	0.73	0.74
	RMSE	0.77	0.63	0.61
DT	*R* ^2^	0.70	0.85	0.95
	RMSE	0.31	0.27	0.17
RF	*R* ^2^	0.72	0.86	0.87
	RMSE	0.34	0.27	0.19
SVM	*R* ^2^	0.73	0.84	0.86
	RMSE	0.28	0.25	0.22
GPR	*R* ^2^	0.74	0.86	0.92
	RMSE	0.32	0.27	0.18
ANN	*R* ^2^	0.85	0.88	**0.97**
	RMSE	0.07	0.06	**0.04**

LR, linear regression; DT, decision trees; RF, random forest; SVM, support vector machines; GPR, Gaussian process regression.

### Evaluation of spectral image correction

**Fig. 5.**
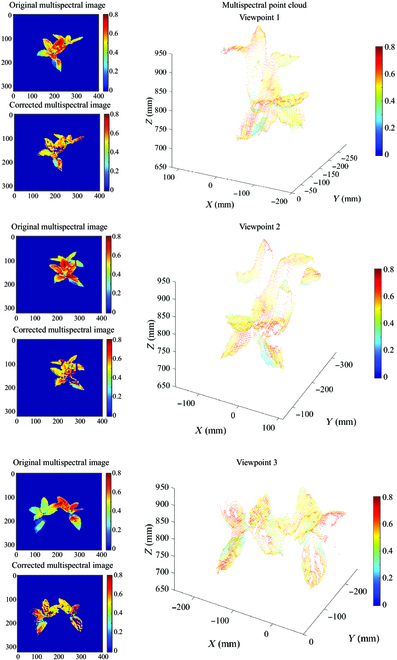
Visualization of multispectral images and point clouds of plants (wavelength of 740.7 nm). Original multispectral images, corrected multispectral images, and multispectral point clouds from 3 different viewpoints are presented in this figure. Spectral textures are displayed in pseudo-color to facilitate comparison. From the difference of the pseudo-color images before and after reflectance correction, the illumination effects were greatly reduced.

Figure 5 shows some examples of multispectral images of plants before and after reflectance correction, as well as multispectral point clouds generated at the wavelength of 740.7 nm. Since the RGB-D images and the multispectral images have been registered in the preprocessing step, the multispectral reflectance rendering was directly adopted to generate a single frame of plant multispectral pseudo-color point cloud. It is clear that the illumination effects were reduced from the difference of the pseudo-color images before and after reflectance correction.

**Fig. 6.**
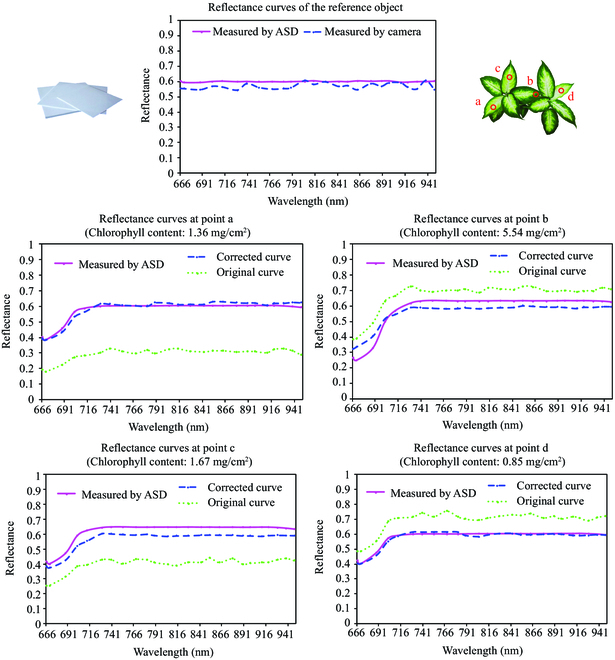
Comparison of multispectral reflectance curves before and after reflectance correction. To facilitate obtaining the reflectance of the hemisphere reference, a flat plate of the same material was selected, and its reflectance was measured using the snapshot multispectral camera and ASD spectrometer. The results are displayed in the first row. Four measured leaf positions were selected as examples to be presented, labeled a, b, c, and d in turn.

Figure 6 presents the comparison of multispectral reflectance curves before and after reflectance correction. The calculated reflectance of reference showed in the first subplot was about 0.58, which was slightly smaller than the measured value of 0.60 by the ASD spectrometer, and the standard deviation between the reflectance of different bands was slightly larger than the measured value of the ASD spectrometer. This indirectly proved that the ASD spectrometer with its own light source was more robust when measuring the spectra of objects. Four of 12 leaf positions were selected as examples to be presented, labeled a, b, c, and d in turn. Points b and d were located higher up in the plant canopy, closer to the light source and at a lower angle of incidence. Points a and c were opposite. Since the chlorophyll absorption curve had larger absorption peaks in the red and blue regions of the visible light band, the true spectral reflectance of point b at 665 to 700 nm was lower. The chlorophyll contents of points a, c, and d were similar, and their true spectral reflectance curves also showed high similarity. However, the differences in the above physiological and biochemical parameters could not be reflected by conventional measurement using the flat reference. In contrast, the reflectance curve obtained by the reflectance correction method proposed in this study effectively reduced the error, retained the variation trend of the original curve, and improved the correlation between spectral reflectance and plant physiological traits.

**Fig. 7.**
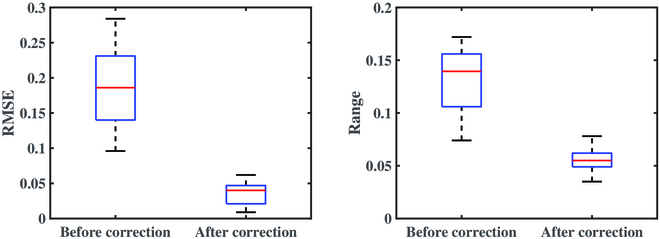
Comparison of RMSE and distance range of multispectral reflectance curves before and after reflectance correction at all measured leaf positions.

Figure 7 shows comparison of RMSE and distance range of multispectral reflectance curves before and after reflectance correction at all measured leaf positions. The same leaf position could appear in different viewpoints, leading to higher RMSE of original spectral reflectance curve (RMSE = 0.17). The average RMSE after reflectance correction was 0.04, significantly lower than original one. The Euclidean distance results between pairs of curves show that the spectral reflectance of the same leaf position before reflectance correction was different because of the difference in illumination effects caused by varying viewpoints. The distance range was generally greater than 0.1, with an average of 0.14. Undoubtedly, the reflectance difference caused by the physiological traits of the plant itself would be confounded with those caused by illumination effects, thereby increasing the analysis difficulty of correlation between the plant physiological traits and spectral responses. After correction, the distance range of the multispectral reflectance curves at the same leaf position was reduced to within 0.1 in general, and the average value was 0.06, which was slightly larger than the measurement error and standard deviation of the multispectral camera. Together, the illumination variation due to the difference in plant morphology at each viewpoint introduced systematic errors, which could be reduced by this reflectance correction method as the results suggest.

### Comparison between search-based and model-based methods

**Fig. 8.**
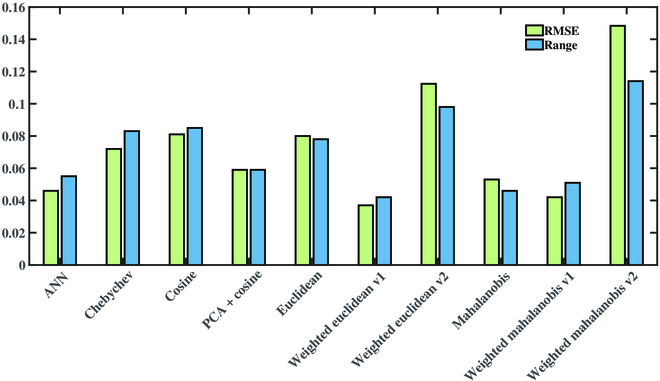
Comparison of average RMSE and distance range of multispectral reflectance curves among using model-based method such as ANN and search-based method with various distance metrics after reflectance correction at the measured positions of all leaves. Euclidean, weighted Euclidean, Cosine, Mahalanobis, Chebychev, Cosine after principal components analysis (PCA), and weighted Mahalanobis are included in the selected metrics. The suffix v1 and v2 stand for both descending and ascending weighting, respectively.

**Fig. 9.**
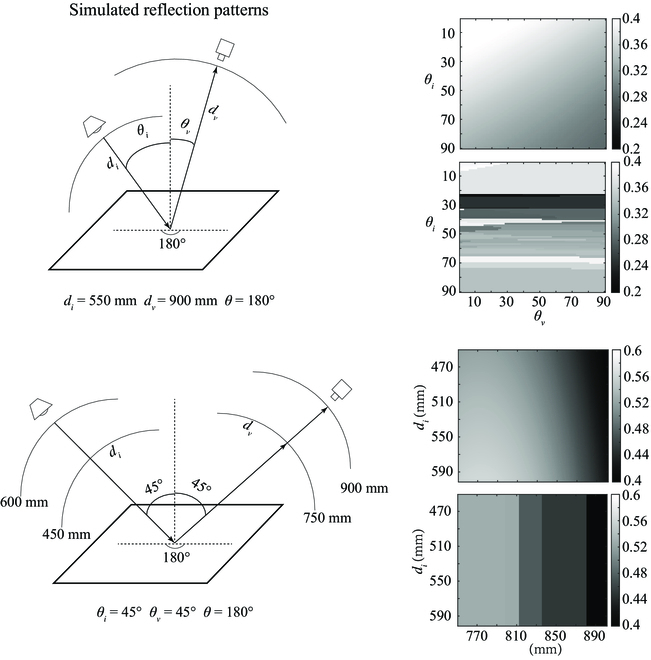
Simulations of the normalized DN values of the references for different incidence and observation cases. The left side showed 2 different incidence and observation cases, the top right side within each row showed the simulated normalized DN values of the references using the trained ANN model, and the bottom right side within each row showed the results obtained by searching in the DN spectra database. The results predicted by search-based method show obvious blocky areas because of the low-resolution sampling while model-based method predicted gradient ones.

Figure 8 presents the evaluations of plant reflectance correction among different search-based methods and ANN model-based method. The weight of the weighted Euclidean distance is the relative importance of the inputs predicting the outputs using random forest-based feature importance ranking. The specific values are 0.82, 0.19, 1.00, 0.04, 0.16, 0.10, 0.84, and 0.29 in the order of *d_i_*, *d_v_*, *θ_i_*, *θ_v_*, θ, *x*, *y*, and *z*. The weight of the weighted Mahalanobis distance is the relative contribution of the principal component variance to the overall variance. The specific values are 1.00, 0.70, 0.38, 0.02, 0.00, 0.00, 0.00, and 0.00 in the order of principal components 1 to 8. We used v1 and v2 to represent the descending and ascending weighting, respectively. Taking feature importance as an example, descending order indicates that important features are given more weight, while ascending order is the opposite. Among the metrics, the descending-weighted Euclidean distance yielded the best correction with the lowest average RMSE and distance range, even outperforming the ANN model. Figure 9 presents simulation results of search-based and model-based methods in different incidence and observation cases. The results predicted by search-based method show obvious blocky areas, while model-based method predicted gradient ones. The above 2 methods were run in MATLAB 2020a software (MathWorks, Natick, MA, USA) on a computer with Intel Core i7-9850H @ 2.60 GHz, Windows 10 64-bit operating system and 64 GB of ECC RAM. The average processing time of the 5-input ANN model prediction and the DN spectra search for a single image were 2.4 and 12.4 min, respectively. It could be seen that the method of training model for predicting the DN spectra of the references was superior to the method of searching in the DN spectra database in terms of prediction efficiency. Overall, with better efficiency and acceptable accuracy loss, the model-based method outweighs search-based method in practicality.

## Discussion

In this section, we will focus on a few systematic errors in the experiment from several aspects. Overall, it appears that the systematic errors come from the idealization of the illumination effects and methodological flaws. The light propagation is highly influenced by the complex canopy structure, yet our previous assumptions about leaves and references could not fully explain this interaction. Moreover, sampling resolution, feature extraction pipeline, and model selection can also make a considerable difference in correction. These issues are discussed in detail in the following subsections, along with potential ways to improve them.

### Illumination effects

Illumination effects, defined as illumination inhomogeneity in the image caused by the interaction of uneven light distribution and complex plant structures, can be classified into one or a combination of several scenarios such as occlusion, multiple scattering, diffuse reflection, specular reflection, etc. Compared to leaf-scale imaging, plant-scale imaging suffers from more severe leaf shading, where the dense canopy and curled leaves blocked incident and reflected light. Figure 5 shows that there were regions of low spectral reflectance (blue or green) on leaves both before and after reflectance correction. However, empirical studies have shown that the reflectance of plant leaves in the near-infrared band is generally around 0.6 (red or yellow), which means that our correction method did not work well on the illumination effect caused by occlusion. The spectral response of this region was formed by the combination of ambient light diffuse reflection and transmission [29]. It is not possible to simulate the occlusion by varying the position of the references, as they were only used to simulate the reflection.

Multiple scattering refers to the phenomenon where light scattered from a leaf is rescattered from neighboring leaves before reaching the sensor. This effect was not obvious on the plant we used because of the low complexity of the canopy structure. Considering that the close placement of the hemisphere references might lead to multiple scattering, we had special control over the number, size, and placement distance of the hemisphere references. However, for crops such as maize and wheat, bushy and clumped leaves close to the main stems can cause multiple reflections of incident light on them to form local bright areas, thus increasing measurement errors [28].

Specular reflection occurs when rays of light are reflected off a very smooth surface and therefore reflect at the same angle. To reflect ultraviolet rays, resist diseases, and prevent nonstomatal water loss in plant tissues, there is usually a waxy layer outside the epidermal cells of plant leaves that increases the specular reflection component, so that the common leaves are not isotropic standard Lambertian [30]. Although adequate water supply and low light conditions provided by indoor semihydroponic cultures can reduce the thickness of the waxy layer and weaken the specular reflection, the extent of the weakening was not quantified. Similarly, the matte surface of the reference is also not a standard Lambertian. In contrast, we used the division of the DN values to calculate the reflectance based on the standard Lambertian assumption for both, which inevitably introduced errors. There are some outlier pixels in Fig. 5 that are obviously defy common sense and have been segmented by thresholding, such as negative values, positive values close to 0, and values greater than 0.8. One reason is the high brightness formed by specular reflection. However, using threshold segmentation or cluster segmentation to remove these pixels results in the loss of spectral information masked by bright regions [31]. Designing a spectral index is a promising approach to not only reducing the influence of illumination effects but also accurately correlating plant physiological and biochemical parameters. However, the original spectrum cannot be obtained using this method [32].

**Fig. 10.**
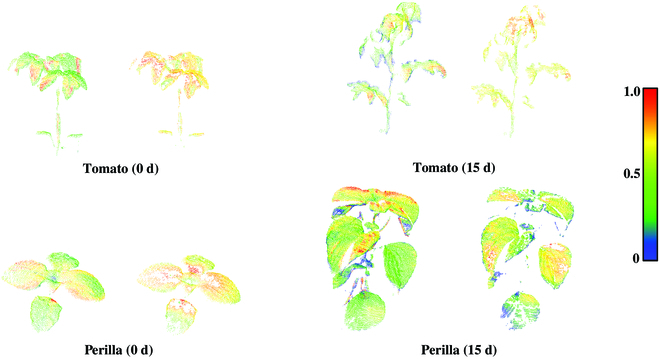
Visualization of the 3D multispectral point clouds of perilla and tomato plants (wavelength of 740.7 nm) before and after correction for different periods. The left side of each subplot shows the 3D multispectral point cloud before correction, and the right side shows the 3D multispectral point cloud after correction.

**Fig. 11.**
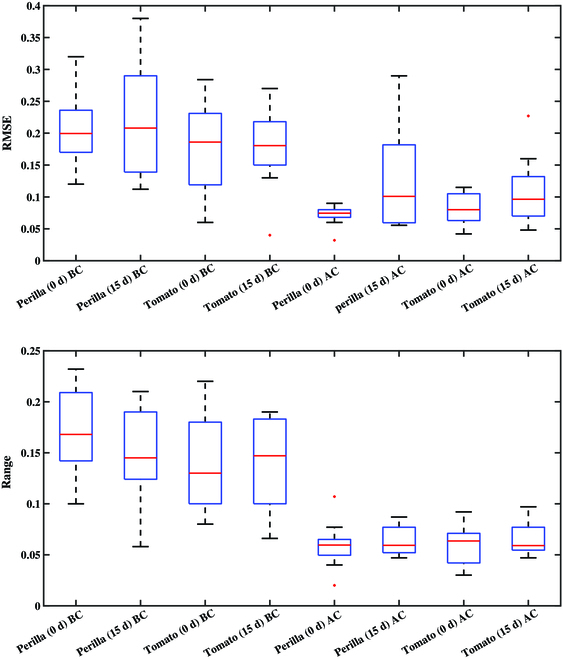
Comparison of RMSE and distance range of multispectral reflectance curves before correction (BC) and after correction (AC) at all measured leaf positions of perilla and tomato plants at different periods.

In conjunction with the above analysis, we presented a case study to quantify the adaptability of our correction method to differences in illumination effects due to species and temporal variation. We corrected the reflectance of tomato and perilla plants at 15-d intervals using the same method as in Materials and Methods. Figure 10 shows the 3D multispectral point clouds of perilla and tomato plants before and after correction for different periods. Figure 11 shows the quantification of the correction. We noted that the differences in leaf physiological traits, such as waxy layer thickness, resulted in an increase in the average RMSE of the corrected reflectance curves compared to the semihydroponic plant. In addition, the corrected RMSEs of the 15-d plants were all significantly large. The temporal effects are mainly reflected in plant height and canopy complexity, which vary across species. For 0-d perilla, the flat leaves and low canopy complexity ensured a better correction. However, the increase in canopy complexity of perilla was much greater than that of tomato. Therefore, the reflectance correction of 15-d perilla was more susceptible to occlusion and multiple scattering. In contrast, except for a small amount of occlusion, the source of RMSE outliers in tomato was mainly the complex leaf structure. The normal estimation of tomato leaves with smaller area was greatly affected by the resolution. It was also susceptible to leaf curling due to environmental factors such as moisture, light, and temperature, further exacerbating the difficulty of correction. Conclusively, we recommended using our correction method for plants in growth stages with low canopy structural complexity and flattened and broad leaves.

All 3 of the above issues regarding illumination effects can be improved by automated acquisition of multipose data. For example, a collision-free path suitable for data acquisition can be automatically planned by the pose information provided by the sensor-equipped robotic arm, thus greatly expanding the quantity and quality of the dataset [33]. As a result, stereo references with the same 3D shape as the original plant can be simulated more accurately, and the light field distribution can be determined for high accuracy reflectance correction [34].

In addition to adaptive multipose imaging, specular reflection can also be eliminated by adopting a BRDF model that can account for both diffuse and specular reflections and its parameterization. It has been reported that the Cook–Torrance BRDF introduced new ideas that only those microfacets oriented toward ***h*** (halfway vector) contribute to the reflection [35]. In this approach, reflection is described by a combination of the diffuse and specular parts with parameters *k_d_* and *k_s_* controlling the respective reflection energy. These parameters are considered a characteristic of the material (e.g., leaves or references in our case). The diffuse term and specular term are represented by a classic Lambertian reflection and a Torrance–Sparrow model, respectively. Accordingly, parameters such as *k_d_*, *k_s_*, and ***h*** can be added to our proposed model as future work dedicated to solving specular reflection.

### Sampling resolution

Another reason for outlier pixels obviously contrary to common sense in Fig. 5 is that ANN models with different inputs had errors in their predictions for very small true values, resulting in predictions of some reflectance of reference close to 0 or negative. The limited sampling resolution is an important factor, which can be adjusted by varying the spacing of the hemisphere references during data acquisition. Since the sampling resolution cannot be increased indefinitely, a balance of time spent and precision is required. Although a suitable sampling resolution was selected through pre-experiments, the predictions of the ANN model still had errors. It seems that ANN has been bottlenecked as it is difficult to further improve the anomalies by increasing the sampling resolution. To ensure the accuracy of the results, we used threshold segmentation to remove outliers rather than image smoothing or bilinear interpolation for adjustment. However, omitting outlier pixels reduces the information in plant digital twins, which, in turn, diminishes the accuracy of plant organ recognition, segmentation, and visualization. The search-based model is known to have a good advantage in accuracy, which may have the potential to achieve a better performance for a given resolution. In addition, increasing the sampling resolution will be helpful in improving the discontinuity of the existing results. Thus, the search-based method is more favorable in cases where there is a demand for accuracy and no limit to efficiency.

### Normal vector estimation

The errors in the calculation of coordinates and distances in feature extraction pipeline mainly come from the RGB-D camera. However, the calculation of angles is highly dependent on the normal vector estimation. Points contained within a sliding window are adjacent in the image, but not necessarily in space, causing a bias in estimation of the normal vector, particularly evident in the edge pixels. Another factor that affects the estimation is the size of the sliding window. A larger sliding window will cause loss of leaf texture details. Therefore, proper hyperparameters need to be selected according to the complexity of the plant structure (e.g., small, curly leaves require smaller sliding windows).

### Model interpretability

When it comes to practicality, the model-based method such as ANN outweighs search-based method for its better efficiency and acceptable loss of accuracy. The validity of such models is mainly based on feature selection, which is also a major focus for selecting models with good interpretability. BRDFs are indeed the best simulation and have long been used to calculate bidirectional reflectance of plant canopies [36]. However, the theoretical BRDF is extremely difficult to use because of the complex expression, high computational effort, and unknown model applicability. Empirical BRDF (e.g., Phong [37] and Blinn–Phong [38] reflection model) mainly aims to provide simple formulation specifically designed to mimic a kind of reflection without considering the physics law behind it. Experimental BRDF can be acquired using a gonioreflectometer that mechanically varies light source and sensor positions. Accordingly, our ANN model can somehow be viewed as an efficient experimental BRDF that directly learn the mapping between features and reflectance. High accuracy models place high demands on the ability of the selected features to interpret the illumination condition. The results of our study show that increasing the number of input features could effectively improve the model performance. The 8-input ANN model, achieving the best result, included features of 3D coordinates, relative distances, and angles, which more comprehensively described the relative positions of light sources, hemisphere references, plants, and sensors. The 5-input ANN model, despite taking distance and angle factors into account, could not infer the 3D coordinates of plants that contained more information in reverse. The 3-input ANN model, only including the 3D coordinates of plants, had the weakest interpretability because the disordered input of point clouds made it impossible for ANN model to learn topological relationships between point clouds, on which the angle calculation relied to find neighborhood points. At present, there are point cloud deep learning algorithms that do not rely on artificial feature selection, which are mainly used for the segmentation of different plant organs [39]. It is foreseeable that these algorithms can directly achieve the end-to-end reference spectra prediction of plants and will obtain better prediction accuracy and interpretability.

## Conclusions

This study shows that it is promising to use stereo references to correct plant spectra and generate high-precision 3D multispectral point clouds of plants. We improved the existing method for generating plant 3D multispectral point clouds from 2 aspects. One is to perform the SURF-Demons to improve the registration of close-range RGB-D images and multispectral images. The other is to design a sampling and a modeling method for spectral DN values of the references to achieve the reflectance correction at the plant level. The results show that the SURF-Demons is competent and achieves better results than the classical rigid registration models. The ANN model achieves a good prediction of the DN values of the hemisphere references based on the plant experimental BRDFs. Overall, our method can be used to obtain accurate 3D multispectral point cloud model of plants in a controlled environment. The models can be generated successively without varying the illumination condition. Future studies will be performed on improving the feature extraction of the point clouds for more complex reflection (e.g., non-Lambertian bodies) correction using deep learning algorithms.

## Data Availability

The data are freely available upon reasonable request.

## Supplementary Materials

Supplementary 1 Fig. S1. Schematic diagram of BRDF model. Fig. S2. Results of the plant multimodal image registration process in each stage. Fig. S3. Visualization of 3D light field feature maps of plants and hemisphere references. Fig. S4. Performance of ANN models with different combinations of 3D light field features trained as inputs for spectral DN values prediction.
