# Supplementary material for: Generating 3D Multispectral Point Clouds of Plants with Fusion of Snapshot Spectral and RGB-D Images
Source: Plant Phenomics. 2023 Apr 3;5:0040. doi: 10.34133/plantphenomics.0040 (PMC10069917; doi:10.34133/plantphenomics.0040)
Supplement: Supplementary 1 — Fig. S1. Schematic diagram of BRDF model. Fig. S2. Results of the plant multimodal image registration process in each stage. Fig. S3. Visualization of 3D light field feature maps of plants and hemisphere references. Fig. S4. Performance of ANN models with different combinations of 3D light field features trained as inputs for spectral DN values prediction. [file plantphenomics.0040.f1.docx]

Supplementary Materials

Figures S1 to S4

**
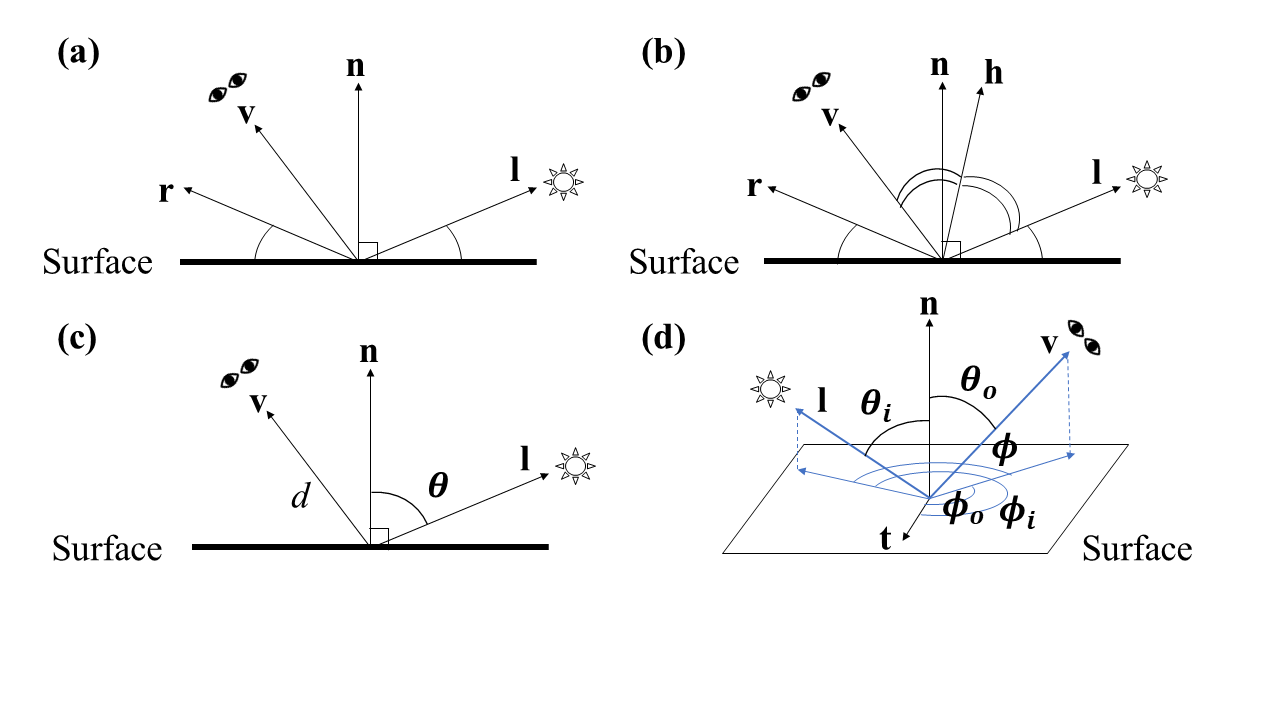
**

Figure S1. Schematic diagram of BRDF model. Here, $\theta_{i}$ and $\theta_{o}$ are zenith angles of the incident light and the observation direction, $\phi_{i}$ and $\phi_{o}$ are azimuthal angles of the incident light and the observation direction, and $\phi$ denotes the angle between the projection of the incident light direction vector and the projection of the observation direction vector on the surface.


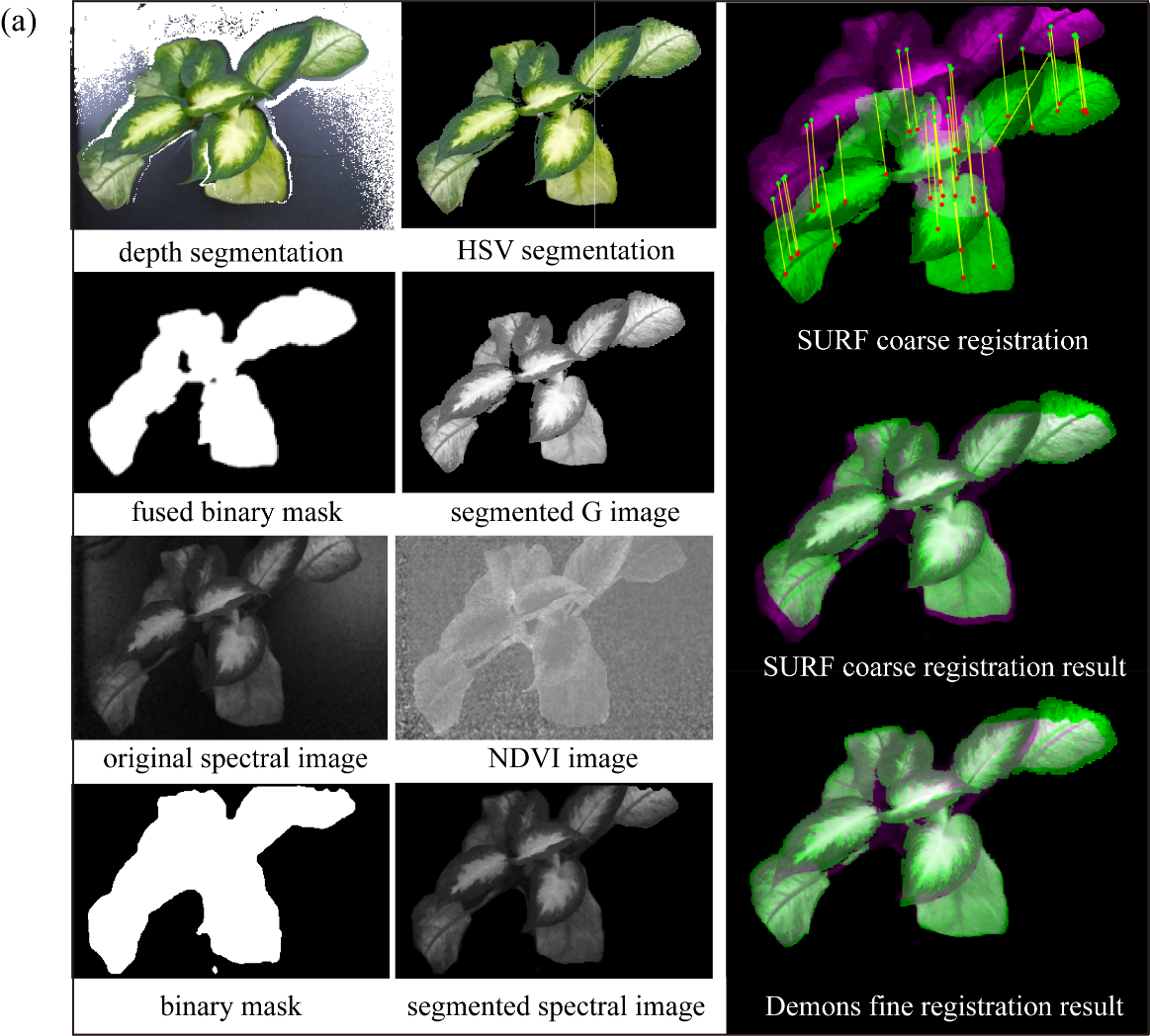


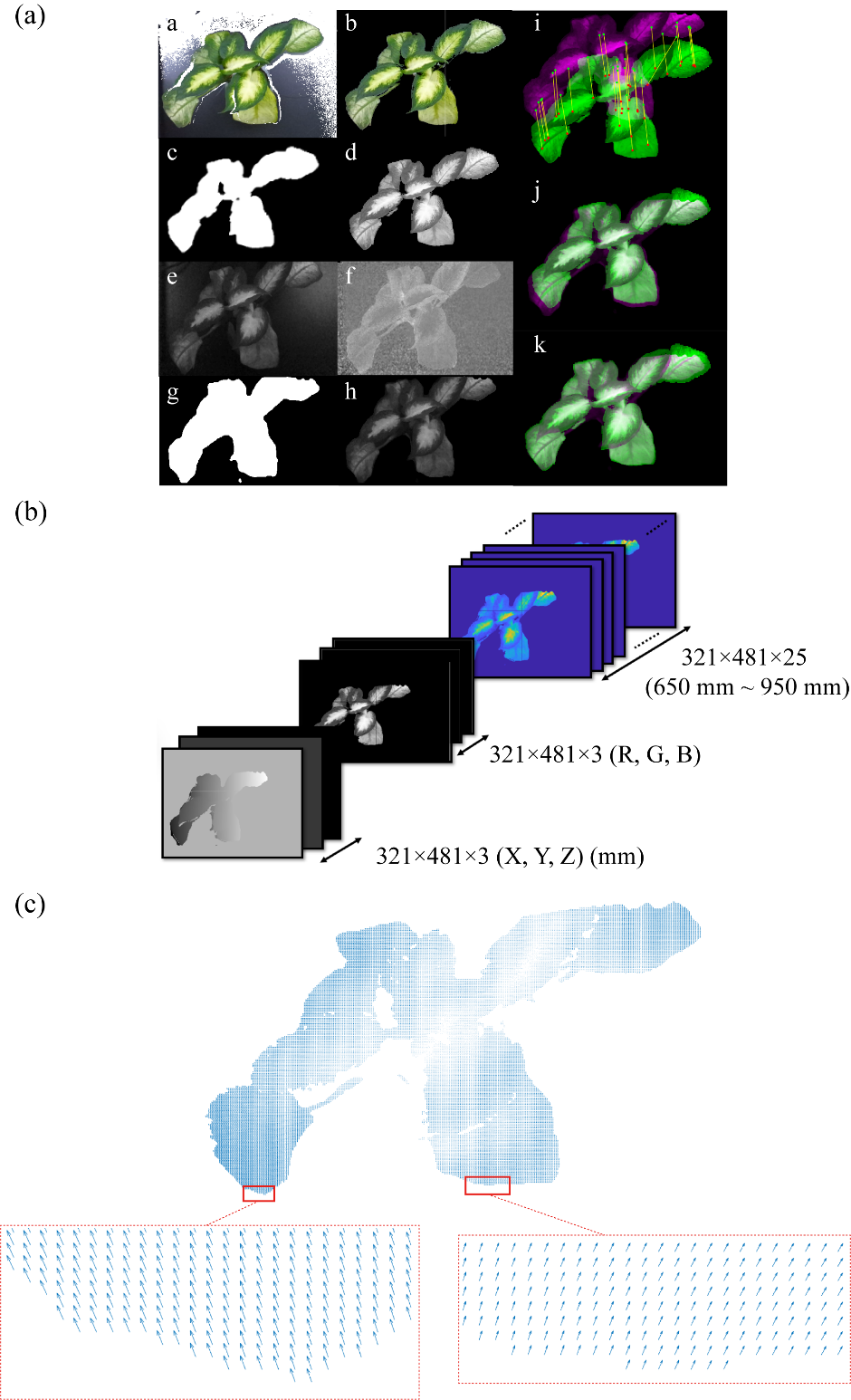


Figure S2. Results of the plant multimodal image registration process in each stage. (a). Results of plant image preprocessing and SURF-Demons registration. Threshold segmentation was performed for both RGB-D image and spectral image using indicators of hue, saturation, value (HSV) and normalized difference vegetation index (NDVI). Then the G image and spectral image (740.7nm) was registered. In this figure we presented an example of the whole preprocessing procedure. (b). The multimodal image cube obtained after registration, which contains the registered depth images, RGB images and 25-band multispectral images. (c). Visualization of the deformation field of plant pixel positions after fine registration by Demons algorithm. Among them, we highlighted two different trends located at two leaf positions and found that both matched the desired varying direction.


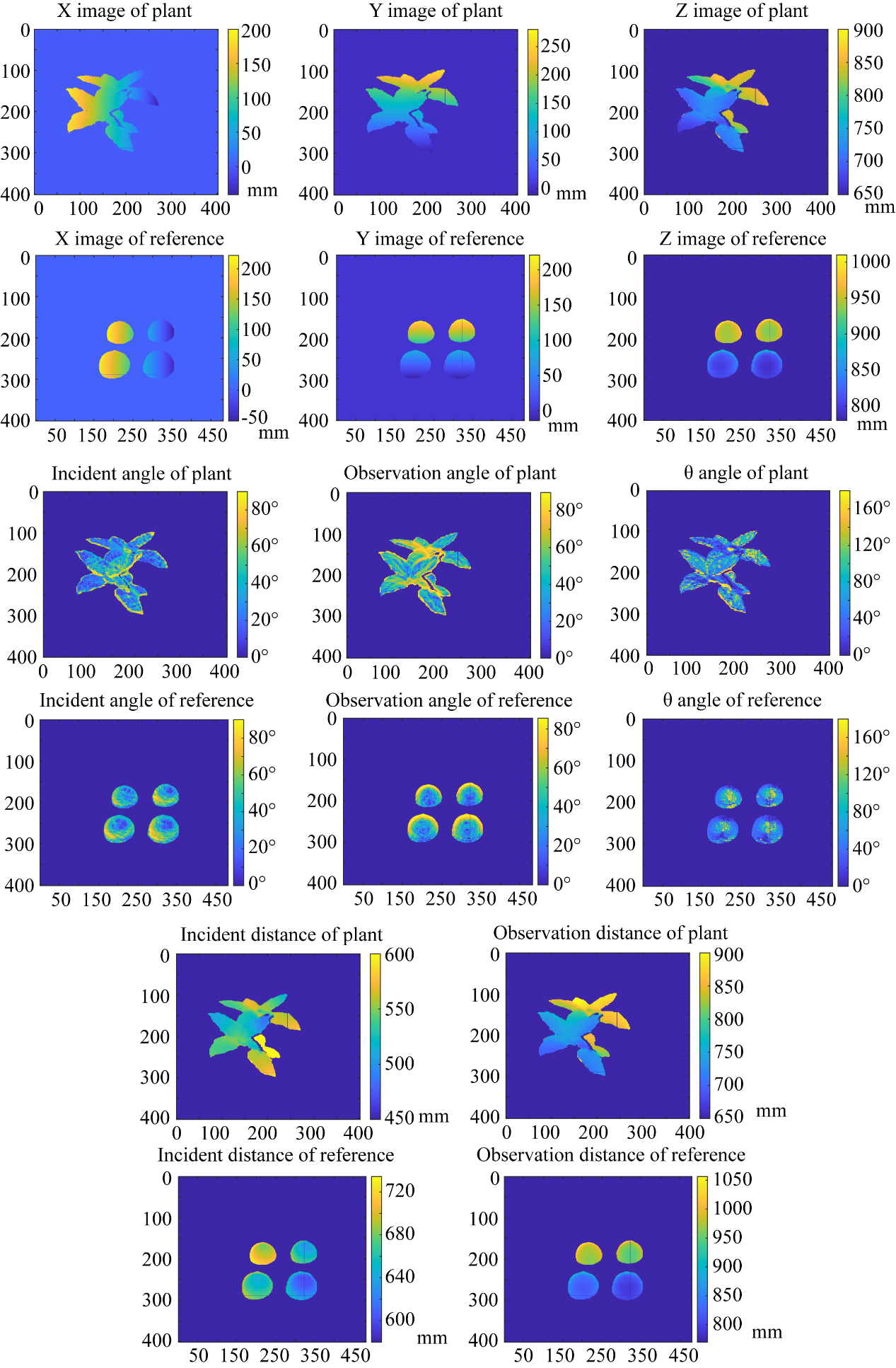


Figure S3. Visualization of 3D light field feature maps of plants and hemisphere references. These features include ${x,y,z,\theta}_{i},\theta_{v},\theta,d_{i},d_{v}$, representing the spatial position, incident and observation directions, incident, and observation distances, respectively. The input of the ANN model is a one-dimensional vector reshaped from these feature maps.


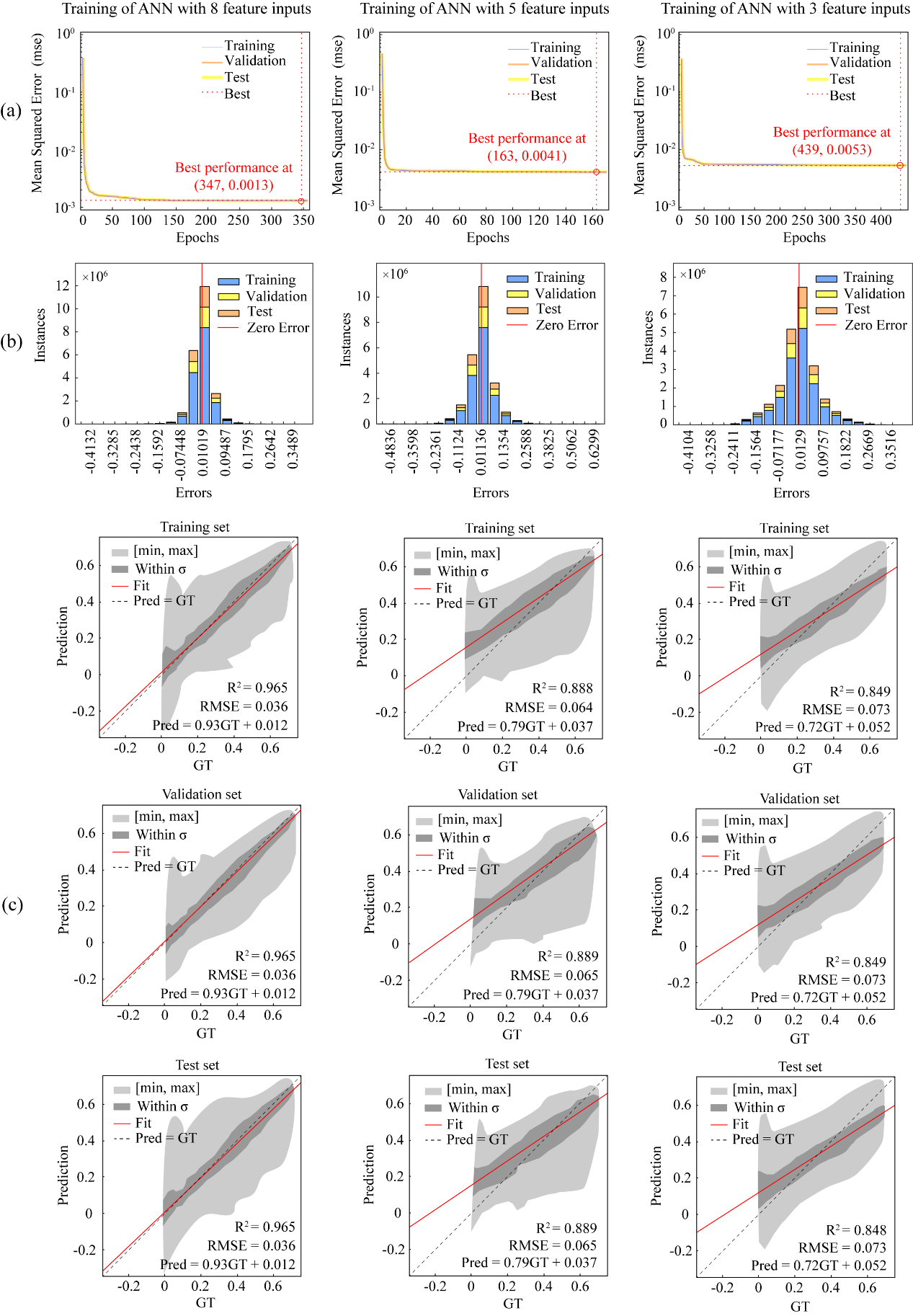


Figure S4. Performance of ANN models with different combinations of 3D light field features trained as inputs for spectral DN values prediction. Each column in order represents 8 inputs $(d_{i},d_{v},\theta_{i},\theta_{v},\theta,x,y,z)$, 5 inputs $(d_{i},d_{v},\theta_{i},\theta_{v},\theta)$, and 3 inputs $(x,y,z)$. (a). The curve of the loss function changing with the training epoch. With adequate training, the mean squared error of the models with different inputs in order were reduced to 0.0013, 0.0041, 0.0053. (b). The error distribution histogram of the DN values of the references predicted by different models. According to the standard deviation of the error distribution, the 8-input ANN model has the most concentrated error distribution, followed by the 5-input ANN model and the 3-input ANN model. (c). Regression results between predictions and ground truth. Each row in order represents training set, validation set, and test set. Compared to 5-input ANN model and 3-input ANN model, the 8-input ANN model performed the best in R-square, RMSE and standard deviation.
